# Induce defense response of DADS in eggplants during the biotrophic phase of *Verticillium dahliae*

**DOI:** 10.1186/s12870-022-03527-7

**Published:** 2022-04-05

**Authors:** Muhammad Ali, Husain Ahmad, Bakht Amin, Muhammad Jawaad Atif, Zhihui Cheng

**Affiliations:** Department of Vegetable Science, College of Horticulture, Northwest A&F University, Yangling, 712100 Shaanxi China

**Keywords:** Diallyl disulfide, PR-genes, Plant hormones, Phenolic compounds, *V. Dahliae*

## Abstract

**Purpose:**

*Verticillium* wilt is a destructive vascular disease in eggplants. The complex defensive mechanisms of eggplant against this disease are very limited.

**Methods:**

Our work examined the bioactive properties of garlic allelochemical diallyl disulfide (DADS) as potential biostimulants for defense against *V. dahliae* in eggplant seedlings. We, therefore, foliar sprayed DADS on eggplants to study the defense response during the early biotrophic phase of *V. dahliae* (a hemibiotroph).

**Results:**

DADS application significantly increased root peroxidase (POD), phenylalanine-ammonia lyase (PAL) enzyme activity, and reduced H_2_O_2_ levels after 24 h of fungal inoculation. Salicylic acid (SA) in leaves and roots was significantly increased while, the jasmonic acid (JA), indole acetic acid (IAA), and abscisic acid (ABA) levels were decreased. The microscopic examinations of *V. dahliae* infection in roots displayed that the progression of infection was restricted in DADS-treated plants. Depositions of lignin and phenolic compounds such as ferulic acid, *p*-coumaric acid, and caffeic acid content were significantly higher in DADS-treated plants at 48 h post-inoculation. Similarly, the DADS application up-regulated pathogenesis-related (PR1, PR2, and PR5), mitogen-activated protein kinase (MPK1), and lipoxygenase (LOX) genes. Furthermore, DADS-treated plants exhibited a lower disease severity index (23.3% vs. 57.0% in controls), indicating successful defense against *V. dahliae*.

**Conclusions:**

Our findings concluded that the biological function of garlic allelochemical DADS has a prominent role in the higher defense resistance of eggplants during the early infection of *V. dahliae*.

**Supplementary Information:**

The online version contains supplementary material available at 10.1186/s12870-022-03527-7.

## Background

Eggplant (*Solanum melongena* L.) is an economically important crop and is often encountered by pathogenic attacks that adversely affect its yield. In China, eggplant is mainly grown inside plastic tunnels where it is highly vulnerable to pathogenic attack, particularly to *Verticillium dahliae-*caused *Verticillium* wilt. It is a potent soil-borne fungal disease that infects eggplants at any growth stage, with the most prominent symptoms being leaf necrosis, wilted areas distinguished by yellow bronze coloration seen between leaf veins, stunted growth, and vascular discoloration [1, 2]. It infects the plants through the roots, and its resting structure (microsclerotia) can survive in the soil for 14 years, even in the absence of a host under cooler soil conditions [3, 4]. Its complete control is very difficult because the source of infection occurs in the soil and the fungus invades internally throughout the plant body.

Synthetic fungicides are being routinely used in developed agricultural systems to control disease and secure crop yields [5]. However, their residue may cause adverse effects on the environment [6]. Therefore, several horticultural practices are still practiced to ascertain the harm and ineffectiveness posed by chemical pesticide application [7]. Past trials have been attempted on eggplant for variety improvements by resistance induction through trait exploitation in wild varieties with limited success [8]. Subsequently, transformation experiments have been performed by inducing *Agrobacterium*-mediated genetic modification while inoculating seedling explants (hypocotyls and cotyledons) and leaves [9]. Grafting attempts in eggplant and tomato have shown elevated tolerance but still susceptibility towards *V. dahliae* [10, 11]. Lately, miRNA, Mir395 from *Arabidopsis thaliana* elevating sulfate production was found to induce hypersensitivity in eggplants towards *V. dahliae* [12]*.* Nevertheless, these practices are very time-consuming and laborious. In place of chemicals, organic-based biostimulants have become a significant focus for sustainable agriculture. It is both cost-effective and promising for priming defense responses against fungal pathogenic invasions as well as improving crop quality and yield while being safe for the environment and health [13, 14]. Plants allelochemicals or extracts from garlic [15], moringa leaves [16], lemongrass [17], and seaweed [18] were reported to improve plant physiology and activate defense systems against pathogen infections. Moreover, plant-microbe interaction studies also show that antioxidant enzymes like superoxide dismutase (SOD) and peroxidase (POD) are the first lines of defense to scavenge the ROS burden produced within the cells as a result of phytopathogen infections [19–21].

Generally, plants trigger several cellular-level defense mechanisms against pathogen invasion [22]. The plant cell wall is mainly composed of cellulose, pectin, and lignin, which represent the most important semi-permeable physical barriers [23]. The reinforcement of the cell wall occurs through the deposition of lignin and lignin-like phenolic compounds during the plant’s defensive mechanisms [24, 25]. Plants were shown to respond to fungal invasion by rapid accumulation of phenolic compounds such as caffeic and ferulic acid in the cell wall, thereby inducing disease resistance to the fungal pathogen *V. dahliae* [24, 26]. Moreover, these phenolic compounds inhibit pathogenic enzymes, thereby restricting the degradation of the cell walls. Notably, plant cell wall perturbations often lead to altered hormonal production [27–29]. Salicylic acid (SA), ethylene (ET), and jasmonic acid (JA) play a major role in regulating plant defense responses against various pathogens, pests, and abiotic stresses [22, 30]. In addition, high levels of SA and JA accumulation in pathogen-exposed tissues induce the expression of pathogenesis-related (PR) genes, which are markers during the onset of systemic acquired resistance (SAR) [31].

Garlic has been recognized as a medicinal plant with a therapeutic effect through most of human history [32, 33]. It consists of several bioactive compounds, among which allicin, or diallyl disulfide (DADS), is widely known due to its bioactive nature [34]. These primal sulphur compounds are responsible for inhibiting a variety of pathogens, including *Phytophthora capsici*, *Botrytis cinerea*, *Verticillium dahliae,* and *Fusarium oxysporum* [35]*.* Numerous researchers have documented the antimicrobial activities of garlic for ages [36, 37]. However, little is known about its mechanism in soil-borne diseases. The allelopathic potency of garlic-derived compounds has a strong allelopathic influence on the cropping environment and may help to alleviate continuous cropping obstacles in eggplant [38], pepper [39], and cucumber [40]. Moreover, garlic straw containing sulphur compounds was found to be the most effective component in inhibiting *Meloidogyne incognita* growth [41]*.* Recent literature has revealed that garlic allelochemicals influence mitotic gene expression and plant hormonal balance in cucumber and tomato plants [42, 43]. However, very little scientific evidence is available on the use of garlic extract as a priming inducer against *Verticillium* in eggplants [44]. Nevertheless, the phenolic metabolism and molecular basis of eggplant’s response to *V. dahliae* in a biotrophic phase are still unclear.

This work aimed to investigate the mechanism of induction of resistance on a molecular basis offered by DADS in eggplant against *V. dahliae*. Here we identify induced PR-genes upon pathogen infection, physiological assessment of the plant’s primary and secondary metabolism such as phytohormonal regulation, peroxidase, phenylalanine-ammonia lyase (PAL) activity, H_2_O_2_ production, phenolic compounds, deposition of lignin, and histological observations to monitor fungal invasion in eggplant at a biotrophic phase of *V. dahliae.*

## Materials and methods

### Plant and fungal materials

Seeds of *V. dahliae* susceptible eggplant cultivar ‘Cui Guan’ (bought from Yufeng seeds company, Yangling, China), were surface-sterilized by immersion in 0.1% sodium hypochlorite solution for 1 min and thoroughly rinsed before sowing in commercial seedling medium-provided plastic trays. Seedlings were transferred from trays to pots (12 × 12 cm) after the emergence of true leaves and allowed to grow in a growth chamber with a 16-h-light/8-h-dark duration at 23 °C. The garlic allelochemical Diallyl Disulfide (DADS) was purchased from Sigma Aldrich (Shanghai) and diluted to a final concentration of 0.4 mmol·L^− 1^(foliar applied 25 mL to each plant), which was previously revealed to be optimal for cucumber resistance to downy mildew [45]. The virulent, defoliating *V. dahliae* strain XJ2008 (originally isolated from cotton in Xinjiang Province, China) was obtained from the College of plant pathology, Northwest A&F University, Yangling, Shaanxi, China [46]. The spore suspensions were prepared according to Gayoso et al., (2010) [24], and cultured on a potato dextrose agar medium for 7–10 days at 25 °C in the dark. Conidia’s concentration was finally adjusted to 10^7^ conidia mL^− 1^ before inoculation.

### Bioassay conditions, and fungal inoculation

A bioassay was performed to assess the induced defense response of DADS in the eggplant. At the 3-4th true leaf stage, seedlings were treated with the selected concentration of DADS through foliar application. A 25 mL solution was applied to each plant, and the same amount of distilled water was applied to control plants. After 48 h of DADS application, a fungal suspension of 20 mL was applied as a soil drench, and then plants were shifted to the growth chamber at 26 °C with 85% humidity and a 16/8 h light and dark duration. The experiment used a randomized complete block design with three biological replicates, for a total of 720 plants. Each treatment consists of 60 plants in a single replication. Treatments consist of Control (CK), and Diallyl disulfide (DADS) both with (+) and without (−) *V. dahliae* inoculation. Plant roots were collected at 0, 6, 12, 24, 48, 72, 144, and 240 hpi (hours post-inoculation) of *V. dahliae* for physiological analysis. At 24 and 48 hpi, the expression of PR genes, phenolic compounds, total lignin content, and differences in the fungal infection progression were examined to determine the role of DADS in eggplant defense mechanisms.

### Statistical analysis

The treatments were subjected to one-way analysis of variance (ANOVA) and the differences in means were determined by using statistix software V.8.1, Tukey’s HSD test (*P* ≤ 0.05) was performed. All figures and pictures were created using the *ggplot* function in R 3.6.0 (R-Studio Team., 2019), and Photoshop CC.

### Eggplant growth attributes

Growth attributes were recorded after 14 days of fungal inoculation. Plant height and fresh biomass were recorded using a measuring tape (cm) and a weight balance, and their dry weight was determined by drying the samples at 75 °C until the weight remained constant.

### Measurement of enzymes activities and H_2_O_2_ content

The root samples were collected at 0, 6, 12, 24, 48, 72, 144, and 240 hpi from both control and DADS plants with (+) and without (−) inoculation of *V. dahliae*. Three biological replicates per time point were collected and immediately frozen in liquid nitrogen before stored at − 80 °C for the estimation of enzymes activity.

POD measurement was determined using the guaiacol method, wherein the solution for determining activity was prepared by mixing 50 mL of 0.05 M phosphate buffer (pH 7.8), 28 μL of guaiacol, and 19 μL of 30% H_2_O_2_ (v/v). Then 3.5 mL of the reaction mixture solution was transferred into a 1 cm path length cuvette for absorbance measurement. After introducing 0.5 mL of enzyme extract into the overall mixture, absorbance was measured over 3 min at 30 s intervals using a wavelength of 470 nm. POD activity was expressed as (U·g^− 1^ FW min^− 1^) [47].

The total PAL enzyme was determined using the method of [48]. The root samples were homogenized and the supernatant was subjected to the Beaudoin-Eagan and Thorpe method. The extract was kept for incubation at 37 °C for 2 h in 10 mM L-phenylalanine, 0.5 M Tris-HCl at pH 8.0, and the reaction was halted by adding 5 M HCl. Spectrophotometric analysis was carried out at 290 nm to measure the resulting trans-cinnamic acid after centrifuging the reaction mixture. PAL activity was expressed as milligrams of cinnamic acid formed per gram of protein.

Hydrogen peroxide levels were measured by initially homogenizing root samples in an extraction buffer (Tris-acetate 50 mM, pH 5.0). The supernatant was collected in a clean tube by filtering the mixture through cheesecloth and centrifuging for half an hour at 14,000 g at 4 °C. After discarding the pellet, control and treated plants were quantified for H_2_O_2_ using the xylenol orange method. For this, 500 μL of root extract is mixed with 500 μL of the reaction mixture (500 μM ferrous ammonium sulfate, 50 mM H_2_SO_4_, 200 μM xylenol orange, and 200 mM sorbitol) [24].

### Endogenous phytohormone extraction and purification

Plant endogenous hormones, salicylic acid (SA), jasmonic acid (JA), auxin (IAA), and abscisic acid (ABA), were determined as described by [49, 50]. An internal standard having 2 μL of H2-JA (2 ng/μL), (40 ng of D_4_-SA), 2 μL of d5-IAA (2 ng/μL), and 25 μL of d6-ABA (0.25 ng/μL) was added to each sample (Yuanye Biotechnology Co., Ltd., Shanghai, China). Samples from leaves and roots (fresh) were taken at 0, 12, 24, 48, and 72 h after inoculation of *V. dahliae* (three biological replicates per time point). Briefly, 0.5 g of sample was pulverized in liquid nitrogen, and an extraction solvent of 0.5 mL (isopropanol: DH_2_O: conc. HCl = 2:1:0.002, v/v/v) was added to the samples and shaken for 30 min at 4 °C at 100 rpm. Then, 1 mL of dichloromethane was added, and the mixture was shaken for 30 min. The solvent was concentrated using a rotary evaporator at 45 °C after centrifugation. Finally, the dry residue was re-dissolved in 1 mL of methanol and filtered through 0.22 μm membranes for LC-MS analysis. The samples were quantified using an Agilent SB-C18 column (50 × 4.6 mm, 1.8 μm) with a sample flow rate of 0.6 mL.min^− 1^ in MRM (multiple-reaction monitoring) mode using an Agilent HPLC system 1260 and a mass spectrometer (an AB Qtrap 5500 triple quadrupole) coupled with an electrospray ionization source. Setting conditions were listed in supplementary materials (Table S1).

### Analysis of quantitative real-time PCR (RT-qPCR)

The relative quantitation of pathogenesis-related PR1, β-1,3-glucanase (PR-2), chitinase (PR-3), Thaumatin-like proteins (PR5), lipoxygenase (LOX), and mitogen-activated protein kinase (MAPK1) genes were observed in eggplant roots (at 24 and 48 h with and without inoculation of *V. dahliae*). RNA was extracted using the E.Z.N.A Plant RNA kit (OMEGA) following the manufacturer’s protocol. The RNA obtained was used to synthesize cDNA using a HiFiScript cDNA synthesis kit (CW2582M, CWBIO, China), in accordance with the manufacturer’s guidelines. The real-time fluorogenic quantitative PCR was performed using SYBR-Green III mixture (E104-A001, JIEYI Biotech, China) in a 20 μL volume, in accordance with the manufacturer’s guidelines. The primers of pathogenesis-related genes and the reference genes were listed in Table S2, obtained from the given references [51, 52]. PCR conditions consisted of an initial denaturation step at 95 °C for 10 min, followed by 40 cycles of 15 s at 95 °C, and 60 s at 54 °C. Melting curves were programmed as 15 s at 95 °C and 60 s at 60 °C, followed by a gradual temperature increase to 95 °C at a rate of 0.3 °C s^− 1^. Analysis of relative expression levels was carried out using the 2^−ΔΔCT^ method [53]. The specificity for each primer pair was verified by melting curve analysis, which revealed that each gene had a single amplification peak (Fig. S1). The analysis was performed with three biological replicates and three technical replicates.

### Analysis of phenolic compounds

Phenolic acids (caffeic acid, ferulic, and *p*-coumaric) were determined in eggplant roots at 24 and 48 h with and without *V. dahliae* inoculation (three biological replicates per time point). Approximately, root samples (150 mg of powder) were extracted with 6 mL of 70% methanol followed by sonication at 30 °C for 30 min. The samples were centrifuged for 7 min at 10,000 rpm at 4 °C. The supernatant was concentrated to a final volume of about 1 mL by filtering and vacuum distillation on a rotary evaporator. Finally, 0.22 μm membranes are used to filter samples for LC-MS analysis [26].

### Staining of eggplant roots with WGA-AF488 and fluorescent brightener 28

The fungal invasion during root colonization was examined using Wheat Germ Agglutinin-Alexa Fluor 488 (WGA-AF488) and fluorescence brightener 28 (FB28) (Wuhan Service Bio-Technology Co., Ltd.) staining. At 24 and 48 hpi of *V. dahliae* infection, eggplant roots were stained with WGA-AF488 to visualize fungal structures and FB28 for cell wall visualization. After clearing the colonized roots in pure ethanol, they were incubated in 10% KOH for 2-3 h at 85 °C. The samples were neutralized with 1× PBS buffer (pH: 7.4) and 4-5 washing steps after incubation. Next, the WGA-AF488/FB28 staining solution (1 μg mL^− 1^ FB28, 10 μg mL^− 1^ WGA-AF488; 0.02% Tween 20 in PBS pH 7.4) was vacuum infiltrated into samples for 4 times (5 min each) at 25 kPa using a desiccator. The WGA-AF488/FB28 staining roots were kept at 4 °C in the dark in PBS buffer until microscopic analysis. Stained root samples were imaged with the Nikon Eclipse E100 (Nikon Co., Ltd., Japan) with a Nikon DS-U3 image capture. WGA-AF488 excitation was at 488 nm and detection at 500–540 nm; FB28: excitation was at 365 nm and detection at 450 nm [54, 55].

### Quantification of total lignin content

Cell walls were prepared using a Triton X-100 washing procedure that included, as the last steps, washing with ethanol (3 times) and diethyl ether (also three times) [56]. Total lignin content was determined using the acetyl bromide method as described in [57]. Alkaline nitrobenzene was used to oxidize lignin in cell walls, and acetyl bromide was measured using absorbance at 280 nm.

### Histochemical analysis

Hypocotyl sections were selected at 48 hpi from 5 DADS-treated plants and 5 control plants (with (+) and without (−) *V. dahliae* infection) [58]. For 10 min, cross-sections were incubated in a phloroglucinol solution (2% (w/v) in 95% ethanol) or (95% ethanol as a staining control), then treated with 18% HCl for 5 min and observed directly with a fluorescence microscope (DM2500; Leica).

### Disease index, and leaf chlorosis

The disease index (DI) was determined by grading symptoms on a 0–4 scale (0; no visible disease symptoms, 1: wilted leaves < 25%, especially in older leaves, 2: wilted leaves 25–50%, 3: wilted leaves 50–75%, and 4: wilted leaves 75–100%). The following formulas were used to calculate the disease index and control efficacy: DI = [(Σ disease grades × each grade’s no. of infected plants)/ (total number of plants examined × maximum disease grade)] × 100%, and control efficacy = [(mean DI of control-mean DI of treatment)/ (mean DI of the control)] × 100% [59]. The severity of leaf chlorosis was graded on a scale of 0–4 [0; no symptoms, 1; up to 25% chlorotic leaves, 2; up to 50% chlorotic leaves, 3; up to 75% chlorotic leaves, and 4; up to 100% chlorotic leaves] [60]. Disease symptoms were scored 14 days after infection.

## Results

### Plant growth attributes are affected by the foliar application of DADS

**Fig. 1:**
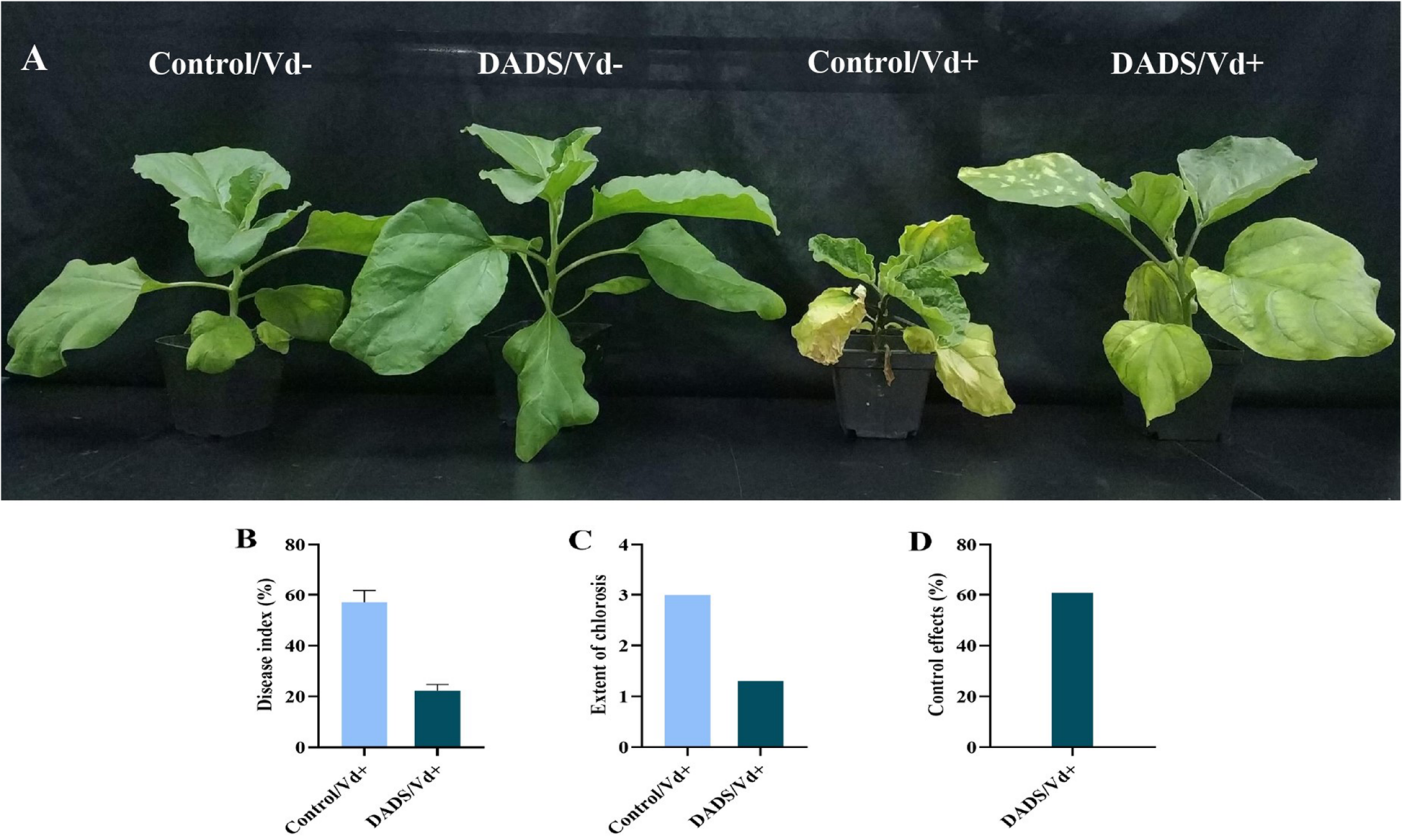
Foliar application of DADS improves the resistance of eggplant against *V. dahliae.* **A**; At 14 days post-inoculation (dpi), the phenotypes of control and DADS-treated plants were photographed. **B**; Assessment of disease Index at 14 dpi. Data are the averages of three biological replications (20 randomly selected seedlings among 60 plants per replicate) in each treatment (±SE, standard error). **C**; Extent of leaf chlorosis symptoms in control and DADS-treated plants at 14 dpi. **D**; Control effect of DADS-treated plants under *V. dahliae* stress

Foliar application of DADS improved the plant growth attributes of eggplant, against *V. dahliae* as shown in Table. 1. DADS treated plants significantly improved plant height, fresh shoot weight, dry shoot weight, and fresh and dry root weight as compared to those of Vd+/control plants. After *V. dahliae* inoculation, DADS-treated plants had the highest plant height (19.4 cm), fresh weight of shoot (42.00 g/plant), and dry weight of shoot (5.727 g/plant) compared to Vd+/control plants. Root attributes such as fresh root (14.59 g/plant) and dry weight (2.855 g/plant) were found to be significantly higher in DADS treated plants than in Vd+/control plants. At 14 dpi, DADS treated plants were healthier as compared to Vd+/control as shown in Fig. 1A. Furthermore, Vd+/control plants showed more chlorotic and necrotic leaves due to pathogen infection. The disease index value was significantly higher in Vd+/control (57.0%) compared to 23.3% in DADS-treated plants (Fig. 1B). Leaf chlorosis was more severe in Vd+/control plants (grade 3) compared with DADS-treated plants (grade 1) Fig. 1C. The Vd+/DADS plants had a 60% controlling effect on disease control Fig. 1D.

**Table 1:** Foliar application of DADS affected eggplant growth attributes at 14 dpi of *V. dahliae*

Treatment		Plant height (cm)	Shoot fresh weight (g)	Shoot dry weight (g)	Root fresh weight (g)	Root dry weight (g)
Control	Vd-	21.4 ± 2.0a	48.57 ± 2.63ab	5.511 ± 0.239a	22.03 ± 1.70a	3.623 ± 0.382a
	Vd+	9.0 ± 1.4b	14.22 ± 2.10c	2.951 ± 0.295b	5.63 ± 0.41c	1.284 ± 0.190b
DADS	Vd-	24.7 ± 1.0a	67.02 ± 4.67a	6.819 ± 0.496a	28.22 ± 2.31a	4.142 ± 0.336a
	Vd+	19.4 ± 1.8a	42.00 ± 3.33b	5.727 ± 0.145a	14.59 ± 1.57b	2.855 ± 0.118a

Treatment values are presented as the mean of three replications (standard error: SE, having 10 plants/treatment in a single replication). Means followed by different letters are significantly different from the controls at (*p* ≤ 0.05) according to Tukey’s HSD test

### Progression of fungal infection is restricted within eggplant roots by DADS

**Fig. 2:**
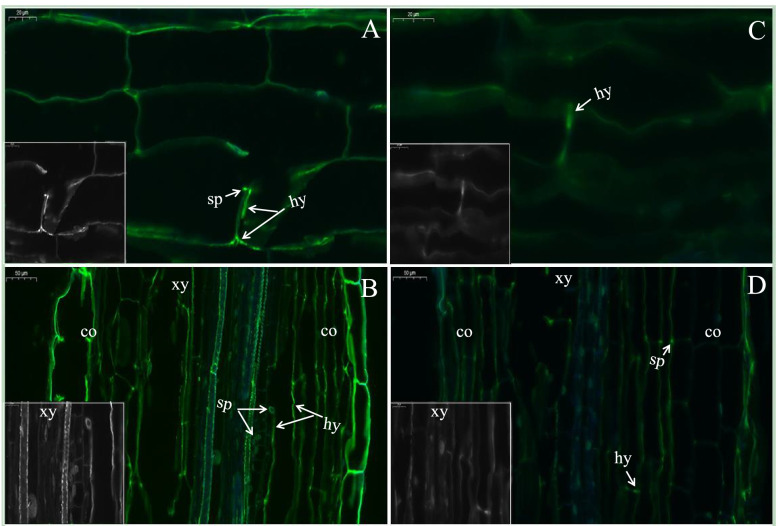
Microscopic observation of the hyphal progression of *V. dahliae* in eggplant roots. Representative images of *V. dahliae* in A to B control (D.W) and C to D DADS-treated plants were taken, at 24, and 48 h post-inoculation (hpi). (A, C; Scalebar: 20 μm) 24 h after inoculation, (B, D; Scalebar: 50 μm), and 48 h after inoculation observation was made on longitudinal section of root hairs, respectively. Roots were cleared and double-stained with WGA-AF488 (green) for fungal structures and fluorescent brightener 28 (blue) for cell wall visualization, and. Co, cortex; hy, hypha; sp., conidia; xy, xylem elements

The conidial germination and hyphal colonization in the root sections were examined at 24 and 48 h after *V. dahliae* inoculation (Fig. 2). In our results at 24 hpi, *V. dahliae* conidia formed germ tubes in control plant roots, whereas in DADS treated plants, the hyphae growth was restricted (Fig. 2, A and C). By 48 hpi, the roots of control plants were highly colonized and formed dense fungal hyphae. In contrast, the cortical cells of the DADS-treated plants showed sparse hyphae (Fig. 2, B, and D).

### Analysis profiles for enzyme’s activities (PAL, POD), and H_2_O_2_ content

**Fig. 3:**
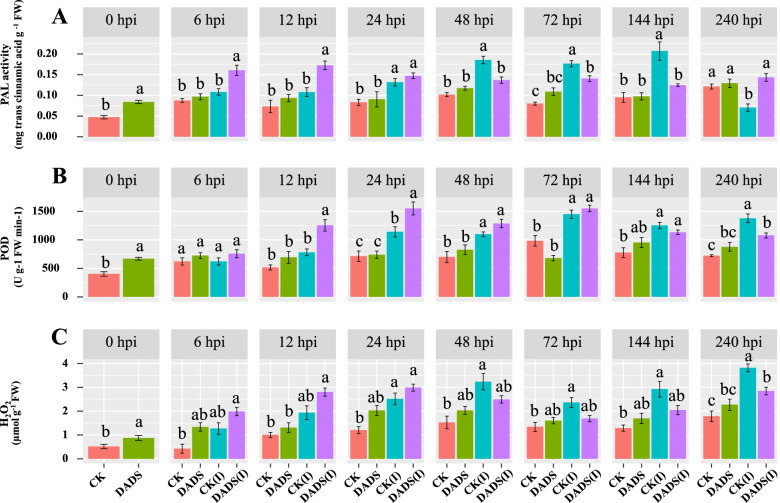
Foliar application of DADS induces phenylalanine-ammonia lyase (PAL), peroxidase (POD), and hydrogen peroxide (H_2_O_2_) contents in the roots of eggplant plants at 0, 6, 12, 24, 48, 72, 144, and 240 h post-inoculation of *V. dahliae.* CK: represents control plants; DADS: diallyl disulfide; CK(I): inoculated control plants; DADS(I): inoculated diallyl disulfide. Data are the average of three biological replicates for each time point. Error bars indicate standard deviations (*n* = 3). Different letters indicate significant differences according to Tukey’s HSD test (*p* ≤ 0.05)

In Vd+/control, the PAL activity dramatically increased from 48 hpi, with the highest activity being observed at 144 hpi, while its activity declined significantly compared to the treated plants at 240 hpi as shown in Fig. 3A. The application of DADS-treated plants showed an increasing trend of PAL activity, with the maximum activity recorded at 12 hpi. However, its activity gradually declined after 24 hpi, and no significant difference was observed afterward (Fig. 3A).

The peroxidase (POD) activity of DADS-treated inoculated plants was maintained at a higher level from 12 hpi to 72 hpi, while its activity declined at 240 hpi. In Vd+/control plants, the POD activity had a delayed increase at 72 hpi; afterward, an increase in POD activity was detected at 240 hpi, which was the maximum as compared to the treated plants (Fig. 3B).

The production of H_2_O_2_ appeared to be stimulated from 0 hpi to 24 hpi in DADS treated plants after *V. dahliae* inoculation, but it accumulated to a relatively low degree from 48 hpi to 240 hpi when compared with Vd+/control plants. At 240 hpi, an increase in H_2_O_2_ content was detected in the roots of Vd+/control plants in comparison to other treatments (Fig. 3C).

### Analysis of hormone accumulation in eggplant tissues

**Fig. 4:**
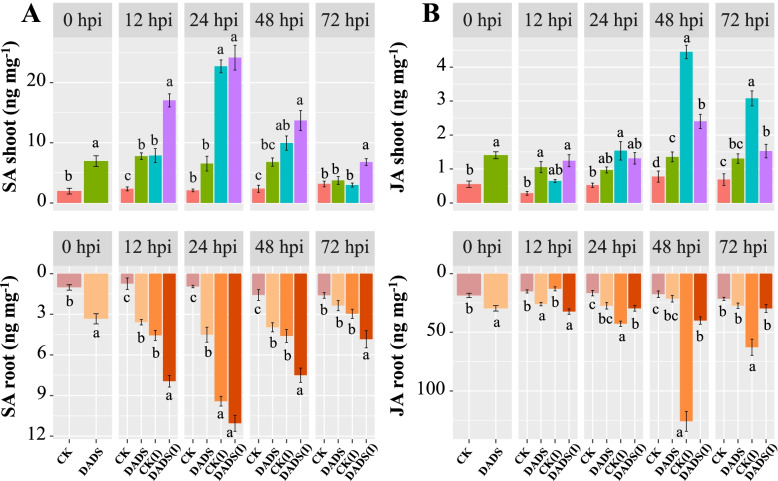
Foliar application of DADS induced salicylic acid (SA) and jasmonic acid content (JA) in the shoots and roots of eggplant plants at 0, 12, 24, 48, and 72 h post-inoculation of *V. dahliae.* CK: represents control plants; DADS: diallyl disulfide; CK(I): inoculated control plants; DADS(I): inoculated diallyl disulfide. Data are the average of three biological replicates for each time point. Error bars indicate standard deviations (*n =* 3). Different letters indicate significant differences according to Tukey’s HSD test (*p* ≤ 0.05)

In shoots at 0 hpi, the levels of SA in response to DADS-treatment were higher by 3.56-folds when compared to Vd−/control plants. While in Vd+/control plants, it increased by 3.35 and 10.93-folds from 12 to 24 hpi, respectively. In contrast to DADS-treated inoculated plants, SA levels were maintained at a higher level from 12 to 24 hpi, resulting in 7.28- and 11.62-folds increases, respectively, when compared to Vd−/controls plants. In root Vd+/control plants, SA concentrations sharply increased by 6.14 and 9.86-folds from 12 to 24 hpi, respectively. Whereas in DADS-treated inoculated plants, the SA levels were at a higher level from 12 to 24 hpi, resulting in 10.70 and 11.56-folds, respectively, as compared to Vd−/control plants (Fig. 4A).

**Fig. 5:**
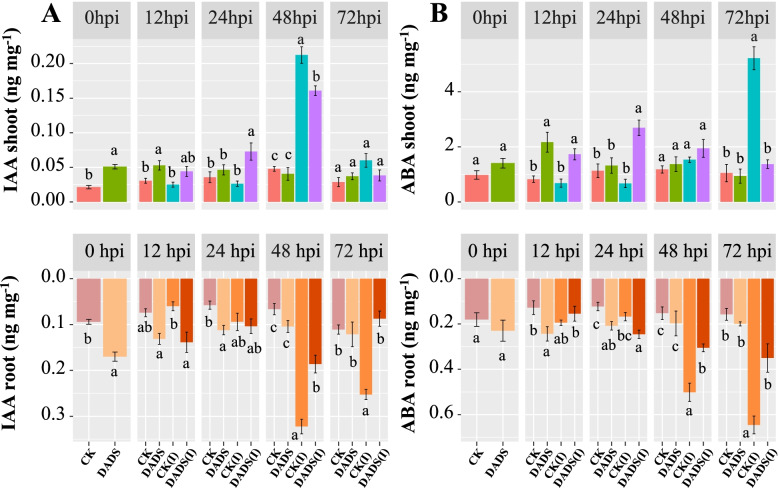
Foliar application of DADS induces auxin (IAA) and abscisic acid (ABA) contents in the shoots and roots of eggplant plants at 0, 12, 24, 48, and 72 h post-inoculation of *V. dahliae.* CK: represents control plants; DADS: diallyl disulfide; CK(I): inoculated control plants; DADS(I): inoculated diallyl disulfide. Data are the average of three biological replicates for each time point. Error bars indicate standard deviations (*n =* 3). Different letters indicate significant differences according to Tukey’s HSD test (*p* ≤ 0.05)

At 0 hpi before the pathogen inoculation, the levels of JA and IAA in both shoot and root were maximum in response to DADS-treatment when compared to Vd−/control plants. As the pathogen infection proceeded at 48 hpi in Vd+/control plants, the JA and IAA levels in the shoots were sharply increased by 5.74 and 4.44-fold, respectively. Whereas in DADS-treated inoculated plants, the JA and IAA levels in shoots were considerably lower at 48 hpi, resulting in 3.10 and 3.36-fold differences, respectively, as compared to the Vd−/control plants. Similar trends were observed in the JA and IAA levels of eggplant roots (Figs. 4B and 5A).

At 72 hpi, significant maximum ABA content was accumulated in Vd+/control plants by 4.98-fold in shoots and 4.09-fold in roots, respectively. Whereas in DADS-treated inoculated plants, the ABA levels were considerably lower by 1.31-fold in shoots and 2.22-fold in roots, respectively, as compared to the Vd−/control plants (Fig. 5B).

### Expression of defense-related genes

**Fig. 6:**
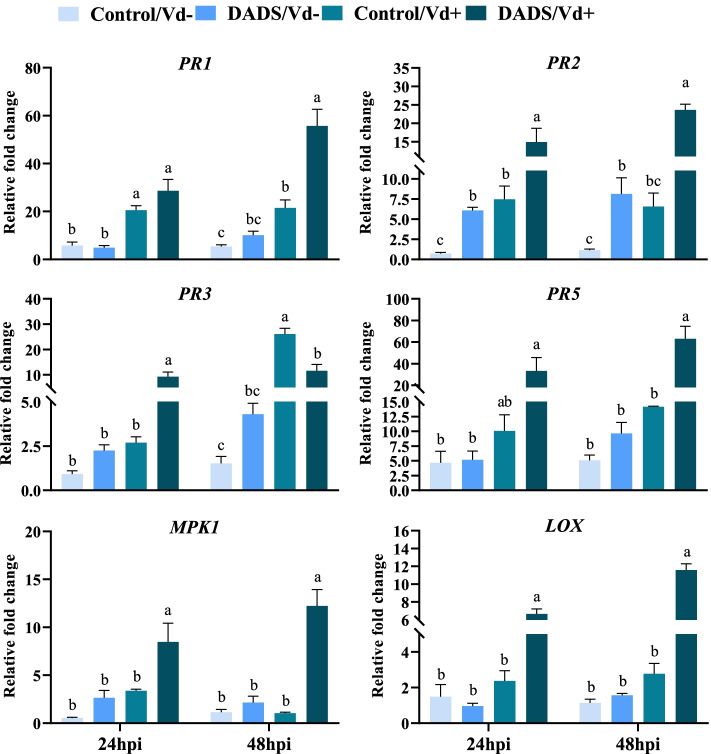
Foliar application of DADS induces relative expression of defense-related genes in eggplant roots at 24- and 48-h post-inoculation of *V. dahliae*. Data are the averages of three biological replicates, and three technical replicates for each timepoint (standard deviation: SD, *n =* 3). Different letters indicate significant differences according to Tukey’s HSD test (*p* ≤ 0.05)

In our results, defense-related genes were induced in DADS-treated plants at 24 and 48 hpi of *V. dahliae* infection in comparison to Vd+/control plants. At 24 h of pathogen inoculation, *PR2, PR3*, *MPK1*, and *LOX* gene expression levels in the DADS-treated plants were significantly increased by 2.0, 3.4, 2.5, and 2.8-folds, respectively, compared to Vd+/control plants. In DADS-treated plants at 48 hpi, the expression level of genes increased significantly by 2.6, 3.6, 4.5, 11.8, and 4.3folds in *PR1, PR2, PR5, MPK1, and LOX*, respectively, when compared to the Vd+/control plants. The *PR3* gene expression level in control plants was upregulated by 2.3-folds as compared to the Vd+/DADS treated plants. The relative expression of the studied genes in Vd−/control and Vd−/DADS-treated plants showed no significant differences. At 24 and 48 h, *PR2* gene expression was significantly increased in DADS-treated plants by 8.3 and 7.2-folds, respectively, when compared to the Vd−/control plants (Fig. 6).

### DADS treated plants have stronger physical barriers and altered phenolic metabolism to induce defense resistance

**Fig. 7:**
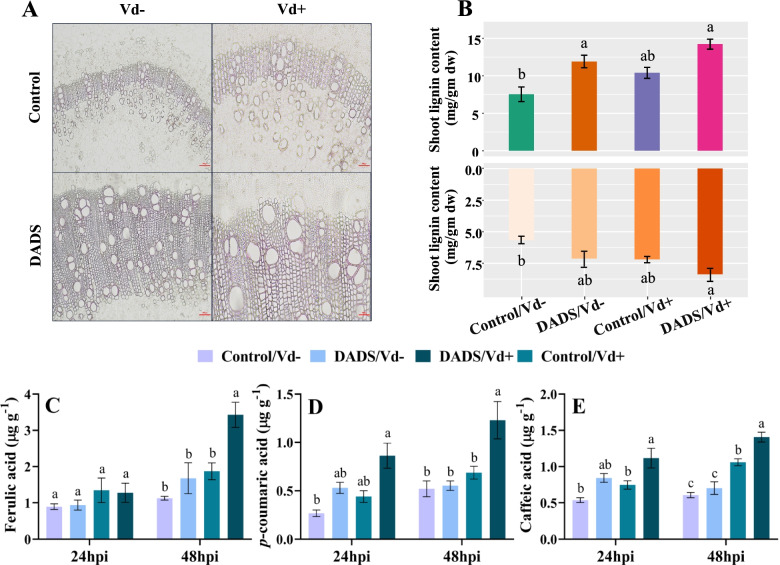
Foliar application of DADS induces lignification and phenolic content to induce defense resistance to *V. dahliae*. (**A**) Histochemical analysis of lignin in the hypocotyl cross-sections of DADS-treated and control plants at 48 h post-inoculation (hpi). Scale bars = 100 px. (**B**) Quantification of the total lignin content in shoots and roots of DADS-treated and control plants at 48 hpi. Comparison of **C**, ferulic acid; **D**, *p*-coumaric acid; and **E**, caffeic acid in the roots of DADS-treated and control plants after 24 and 48 hpi. Data are the averages of three biological replicates for each time point. Error bars indicate standard deviations (*n =* 3). Different letters indicate significant differences according to Tukey’s HSD test (*p* ≤ 0.05)

The phloroglucinol-Hcl staining indicated that at 48 hpi, hypocotyl sections of DADS inoculated plants displayed deeper color in the xylem vessel wall and parenchyma cell walls than those of Vd+/control plants (Fig. 7A). Furthermore, the quantitative analysis of uninoculated plants showed that the levels of lignin in the shoot and root of DADS were higher than those of Vd−/control plants. At 48 hpi, DADS inoculated plants showed inclined levels of lignin in the shoot and root, respectively (Fig. 7B).

Root phenolic contents, i.e., caffeic acid, ferulic acid, and *p*-coumaric acid, showed differences under pathogen infection and DADS application. At 24 hpi, caffeic acid showed a significant increase in DADS treated inoculated plants in comparison to Vd+/control plants. At 48 hpi, caffeic acid, ferulic acid, and *p*-coumaric acid were significantly increased in the DADS treated inoculated plants up to 1.8, 1.8, and 1.3-folds, respectively, then in those of Vd+/control plants. No significant changes were observed between Vd−/control and Vd−/DADS-treated plants, respectively (Fig. 7. C, D, and E).

## Discussion

*Verticillium dahliae* is a soilborne pathogen that causes vascular wilt in eggplant. In our work, the DADS treated plants showed improved physiological parameters, which can be strongly corroborated with our previous work regarding the growth-promoting effect of garlic allelochemicals on eggplant [61], tomatoes [42, 62] and cucumber plants [43]. Previous research revealed that DADS has a dose-dependent effect on cucumber and tomato seedling growth, with lower concentrations (0.01–0.62 mM) promoting growth and higher concentrations (6.20–20.67 mM) inhibiting growth [42, 43]. The allelopathic potential of garlic is based on its biochemical compositions, which include allicin, diallyl disulfides, diallyl trisulfides, etc., and are the major organosulfur compounds in garlic [34, 42, 63]. In our study, the DADS-treated inoculated plants remained comparatively healthier, affirming that the DADS chemical structure is stable and does not denature quickly, which suggests its usage in a long-term agricultural application [64]. Its peculiar and pure form of organosulfur characteristics from garlic extracts aid in decomposing cell wall degrading enzymes of fungus, thereby limiting infection spread in a host cell [65, 66].

ROS levels mark metabolic disparities within the living system, and plants respond to incompatible reactions by expressing resistance genes, stress-responsive proteins, the generation of ROS, the release of phytoalexins and toxins, and localized hypersensitive reactions [24, 67]. Normally, a plant’s ROS activities are maintained in equilibrium due to plant antioxidant and hormonal balance in the cell’s cytoplasm. However, a slight imbalance may trigger a defense system to scavenge the overproduction of ROS. Antioxidant enzymes balance hydrogen peroxide by disintegrating it into water and oxygen molecules, which is a primary defense mechanism against any foreign pathogenic attack. Therefore, any variation in their activity indicates the level of stress or damage [68, 69].

In fungal inoculated plants, the oxidative free radicals must have been produced due to fungal infection that raised POD activity (Fig. 3B). H_2_O_2_ is an oxidative burst marker during plant-pathogen interactions and has been found to slightly shoot up in the roots of DADS treated plants and conversely in control ones, enhancing signaling during hypersensitive reactions and promoting systemic acquired resistance [70, 71]. In our work, the Vd+/control plants revealed an increase in ROS levels. It must be contributing towards growth as oxidative stress hampers plant growth and promotes the appearance of diseased characteristics. Furthermore, any imbalance in ROS production can cause oxidative damage to major macromolecules, i.e., DNA, lipids, and proteins [72, 73].

PAL is involved in phenylpropanoid metabolism, which is a primary step in the metabolism of several phenylpropanoid plant compounds. The DADS-treated plants showed maximum activity at 12 hpi and then gradually declined, while the Vd+/control plants had a gradual increase and declined after 144 hpi. It can be related to elevated H_2_O_2_ levels at 24 hpi. The increased PAL activity in alignment with elevated H_2_O_2_ levels has been demonstrated in resistant tomato plants, while susceptible plants show a delayed rise in PAL activity [24].

Plant hormones are naturally occurring signaling molecules that regulate the growth and development of plants. Pathogen attack activates a hormone signaling network, which limits pathogen invasion [74, 75]. In our results, DADS treatment altered the levels of SA, JA, IAA, and ABA in the shoots and roots of eggplant during *V. dahliae* infection. At 12 to 24 hpi, the rate of SA increase in shoots and roots was significantly lower in the DADS treatment as compared to the Vd+/control plants. The greater fungal biomass accumulation as the disease progresses further and activates additional defense response pathways might be the reason for the rapid increase in SA levels of Vd+/control plants. SA-mediated signaling pathways are predominantly involved in the activation of defense responses against biotrophic and hemibiotrophic pathogens and trigger systemic acquired resistance [22, 76]. In *Arabidopsis*, eggplant, and tomatoes, SA pathways are essential for resistance to *Verticillium* wilt [52, 77, 78].

Higher levels of JA were detected in the roots of DADS-treated inoculated plants at 12 hpi. However, as the infection progressed, lower JA levels were detected in DADS-treated plants, which could activate SA-mediated responses by prolonging *V. dahliae’s* biotrophic phase. Our results are in accordance with the study of Guerreiro et al. (2016) [79] that reported that the JA pathway is activated during the first hours of interaction between grapevine and *Plasmopara viticola*, a biotrophic pathogen. The JA-SA antagonistic interaction between defense signaling pathways provides focused resistance against pathogens at later inoculation time-points [80]. Similarly, IAA levels in both shoots and roots were significantly higher in Vd+/control plants. The higher auxin signaling in Vd+/control plants might be a useful strategy by the pathogen to suppress the SA-dependent defense response during the initial biotrophic phase of *V. dahliae.* As reported by [81], IAA levels were significantly increased in plant leaves with a higher peak at 3 to 4 days after infection with either *Pseudomonas syringae* pv. tomato or *Arabidopsis–Xanthomonas campestris* pv. *campestris*. In addition, IAA and SA signaling pathways also work in an antagonistic way, thereby enhancing susceptibility to biotrophic pathogens [82]. In our results, at 0 hpi, a significant increase in the IAA of DADS treated plants were observed. It might be due to the involvement of DADS in improving cellular mitotic division and root growth expansion in cucumber and tomato seedlings via the regulation of the plant hormone auxin [42, 43]. Furthermore, the higher ABA levels in Vd+/control plants and lower in the DADS treated plants suggest an impaired infection progression. This observation is consistent with the findings of [83], who reported that ABA concentrations increased in *Arabidopsis* leaves at 2 dpi in response to *V. longisporum*. Furthermore, at 6 and 8 dpi, ABA levels were higher in *Arabidopsis* roots infected with *V. longisporum* [84]. In addition, ABA and SA have antagonistic interactions [85], and this antagonism is thought to be responsible for ABA’s increased disease susceptibility against the soilborne fungus *V. longisporum* [86], and *Fusarium oxysporum* [87].

Our results show that the relative expression levels of pathogenesis-related (PR) genes and mitogen-activated protein kinase (MAPK1) levels were up-regulated in DADS-treated inoculated plants. MAPK cascades are important for plant development and response to biotic and abiotic stresses [88]. The up-regulation of the PR genes and MAPK1 levels indicates the biological function of garlic allelochemicals in conferring resistance, thereby SA-mediated activation of SAR, which prevents the spread of infection to healthy tissues. These results are consistent with the previous reports that PR genes, PR1, PR2, and PR5 are the marker genes induced by SA [89]. The increased expression of the chitinase (PR3) gene in Vd+/control plants is consistent with previous findings that JA signaling activation increased the expression of JA marker genes (PR3, PR4, and PR12) during necrotrophic pathogens [90]. Auxin influx triggers the expression of LAX genes [91]. They promote shoot apical meristem and root primordia by reinforcing the auxin-dependent induction of a distinct set of cell-wall-remodeling enzymes [92]. The garlic allelochemical (DADS) function as signaling molecules by regulating plant developmental progression and root growth. DADS up-regulates the expression of LAX5, and IAA2 in tomatoes [42, 93], mitotic-related genes such as *CDKA* (cyclin-dependent kinase A), and *CDKB* (cyclin-dependent kinase B) in cucumber plants [43], which supports the role of DADS at a molecular level.

The rapid advancement of confocal laser scanning microscopy (CLSM) has greatly aided our understanding of *V. dahliae* colonization of various plant roots [94]. Histological analysis of the roots revealed that the progression of *V. dahliae* is restricted in DADS-treated plants. The microscopic observation indicates the resistive nature of DADS in plants, which enables them to restrict fungal invasion. Accumulation of lignin, phenolics, etc. plays a key role in resistance to pathogens invading vascular tissues [95–97]. The increased lignin content in DADS treated plants might be due to the activation of successful resistance to fungal invasion in response to *V. dahliae* attack. A cell wall consisting of higher lignin content is more water-resistant, making it less accessible to enzymes that degrade fungal cell walls [98]. Inoculation with *V. dahliae* caused a significant increase in the lignin content of pepper varieties with varying degrees of resistance response, according to [58]. The abundance of caffeic acid, ferulic acid, and *p*-coumaric acid was significantly increased in the roots of DADS-treated plants with fungal inoculation. The accumulation of these phenolics might also be responsible for the increased resistance against *V. dahliae*. Consistent with other reports, these phenolics are linked to cell wall strength, digestibility, and resistance to pathogen infections [99]. The cross-linking of lignin-polysaccharide interactions in the secondary cell walls of gramineous plants is mediated by *p*-Coumaric acid [100]. Caffeic acid and ferulic acid inhibit the hyphal growth of *V. dahliae* [26]. The fungus *Fusarium oxsporum* encouraged the biosynthesis of caffeic acids and ferulic acid in pepper roots, which strengthened the cell wall to prevent pathogen penetration [101].

## Conclusions

Current research findings are vital in understanding the bioactivity of diallyl-disulfide (DADS) in the defense of eggplant against *V. dahliae* inoculation. Application of DADS induces different defensive responses, resulting in the activation of plant enzymes (POD and PAL), accumulation of phenolic compounds (caffeic acid, ferulic acid, and *p*-coumaric acid), and the reinforcement of cell walls. Here, the intrinsic defense against fungal colonization was significantly higher in DADS-treated plants. By up-regulation of PR genes associated with the hormonal signaling pathways, which indicates that garlic allelochemicals act as a signaling molecule in plant defense responses. Furthermore, these findings lay the foundation that the organosulfur compounds of garlic can act as plant biostimulants, resulting in induced resistance to *Verticillium* infection, particularly in plastic tunnels or glasshouse conditions.

## Supplementary Information

**Additional file 1.**

## Data Availability

The datasets used and/or analyzed during the current study are available from the corresponding author on reasonable request.

## Abbreviations

DADS: Diallyl disulfide
POD: Peroxidase
PAL: Phenylalanine-ammonia lyase
SA: Salicylic acid
JA: Jasmonic acid
IAA: Indole acetic acid
ABA: Abscisic acid
PR: Pathogenesis-related
MPK1: Mitogen-activated protein kinase
LOX: Lipoxygenase
ROS: Reactive oxygen species
H_2_O_2_: Hydrogen peroxide
qPCR: Quantitative polymerase chain reaction
WGA-AF488: Wheat Germ Agglutinin-Alexa Fluor 488
FB28: Fluorescence brightener 28
hpi: Hours post-inoculation
DI: Disease index
