# Supplementary material for: Induce defense response of DADS in eggplants during the biotrophic phase of Verticillium dahliae
Source: BMC Plant Biol. 2022 Apr 5;22:172. doi: 10.1186/s12870-022-03527-7 (PMC8981950; doi:10.1186/s12870-022-03527-7)
Supplement: Supplementary file 1 — Additional file 1. [file 12870_2022_3527_MOESM1_ESM.docx]

# **Table S1: Selected reaction monitoring conditions for protonated or deprotonated plant hormones**

| **PH** | **SM** | **Q1** | **Q3** | **Q2(eV)** | **DP(V)** | **RT** |
| --- | --- | --- | --- | --- | --- | --- |
| SA | − | 136.9 | 92.6 | -10 | -105 | 2.406 |
| JA | − | 207.1 | 58.6 | -6 | -105 | 2.689 |
| IAA | + | 176.0 | 129.8 | 15 | 90 | 2.299 |
| ABA | − | 262.8 | 204.0 | -13 | -105 | 2.434 |

SA, salicylic acid; JA, jasmonic acid; IAA, indole-3-acetic acid; ABA, abscisic acid; PH, plant hormones; SM, scan mode; Q1, precursor ion selected in Q1; Q3, product ion selected in Q3; Q2, collision energy (eV); DP, declustering potential (V); RT, retention time.

Table S2. List of Primers used for the amplification

| Primer name | Primer sequence（5’-3’） | Gene target | Length (bp) | Amplification efficiency | Ta (°C) | Tm (°C) | GenBank/Sol Genomics Network ID |
| --- | --- | --- | --- | --- | --- | --- | --- |
| PR1-F | GTGGGTCGATGAGAAGCAAT | PR1 | 92 | 1.996 | 54 | 56.42 | AB222697.1 |
| PR1-R | TACGCCACACCACCTGAGTA |  |  |  |  |  |  |
| PR5-F | CAAACACCCTGGCTGAATACG | PR5 | 113 | 1.957 | 54 | 56.48 | Sme2.5_30700.1_g00001.1 |
| PR5-R | ACTAGGATTGGTCGGTGCAA |  |  |  |  |  |  |
| GLU-F | AGATTGAGGCTTTATGATCCGA | β-1,3-glucanase | 163 | 1.931 | 54 | 54.78 | KX450413 |
| GLU-R | CTGGCCAGAAATCCTTAACGT |  |  |  |  |  |  |
| MPK1-F | CCTCCGTGGGTTGAAATAC | [Mitogen-activated protein kinase](https://www.sciencedirect.com/topics/biochemistry-genetics-and-molecular-biology/mitogen-activated-protein-kinase) 1 | 159 | 2.015 | 54 | 54.80 | KY861322 |
| MPK1-R | GTCACAACATATTCGGTCATAAAG |  |  |  |  |  |  |
| LOX-F | GGAGGGATCAAACTTCCTCA | LOX | 101 | 1.991 | 54 | 55.40 | AB244527.1 |
| LOX-R | ATTCCTTCACCGTCTGTTCG |  |  |  |  |  |  |
| CHT-1F | AGAGAACAAGGTAGCCCAGG | Chitinase | 150 | 1.978 | 54 | 57.51 | KX450412 |
| CHT-1R | TAAAAGGTCCACTCCGATGGC |  |  |  |  |  |  |
| Actin-F | TGGTCGGAATGGGACAGAAG | ACTIN 1 | 191 | 1.934 | 54 | 57.45 | [XM_009616336](http://www.ncbi.nlm.nih.gov/entrez/query.fcgi?cmd=search&db=nucleotide&doptcmdl=genbank&term=XM_009616336) |
| Actin-R | CTCAGTCAGGAGAACAGGGT |  |  |  |  |  |  |
| GAPDH-F | CCGCTCCTAGCAAAGATGCC | GAPDH | 155 | 1.943 | 54 | 58.00 | [JX524155.1](https://www.ncbi.nlm.nih.gov/nuccore/JX524155.1) |
| GAPDH-R | ACCCTCCACAATGCCAAACC |  |  |  |  |  |  |

Amplicon length (bp), melting (Tm) and annealing (Ta) temperatures are presented


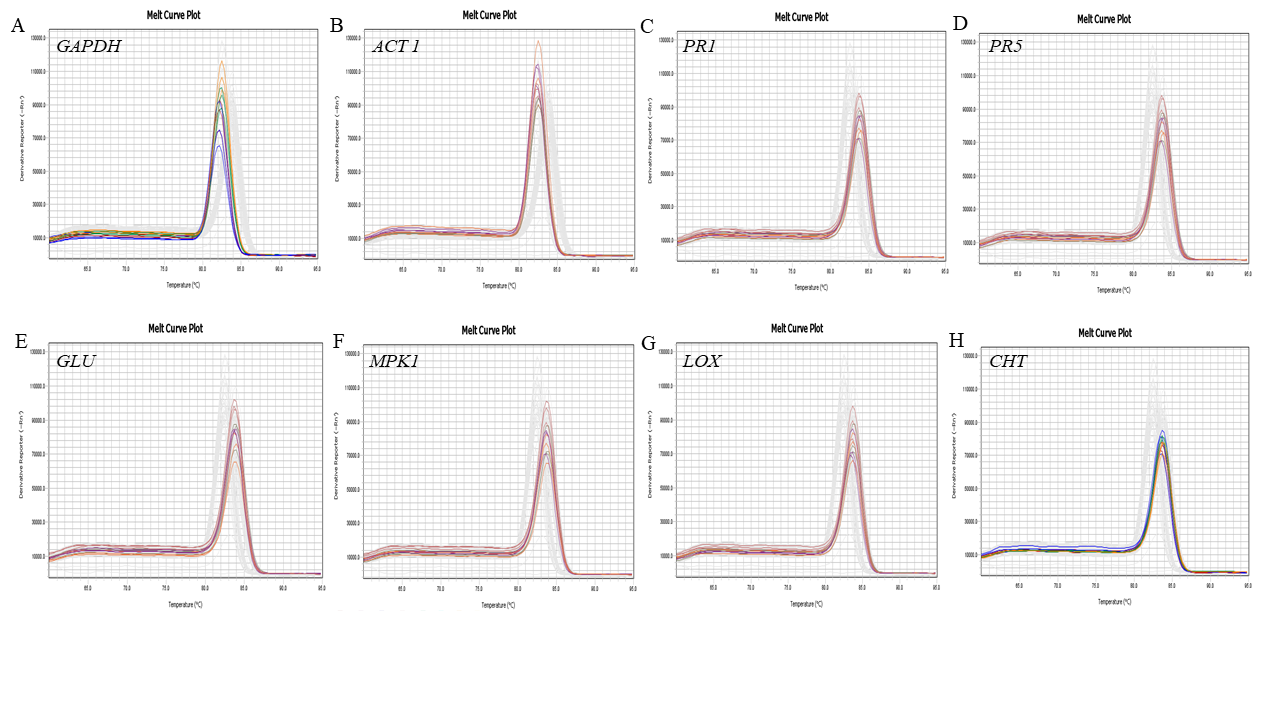
 **Fig. S1. Dissociation curves for the reference and target genes in eggplant.**

Each peak map represents the melting curve of one gene, the peaks representing the melting temperature of each gene, A: *GAPDH*, B: *ACT1*, C: *PR1*, D: *PR5*, E: *GLU*, F: *MPK1*, G: *LOX*, H: *CHT*.
